# A comprehensive overview of the fabrication and testing methods of FRP composite pipes

**DOI:** 10.1016/j.mex.2024.102990

**Published:** 2024-10-03

**Authors:** Senthil Maharaj Kennedy, R.B. Jeen Robert, R. Malkiya Rasalin Prince, G.S. Hikku, M. Kaliraj

**Affiliations:** a Department of Mechanical Engineering, AAA College of Engineering and Technology, Sivakasi 626005, Tamil Nadu, India; b Department of Mechanical Engineering, Sri Krishna College of Technology, Coimbatore 641042, Tamil Nadu, India; c Division of Mechanical Engineering, Karunya Institute of Technology and Sciences, Coimbatore 641114, Tamil Nadu, India; d Faculty of Allied Health Sciences, Chettinad Hospital and Research Institute, Chettinad Academy of Research and Education, Chennai 603103, Tamil Nadu, India

**Keywords:** CFRP composite, Filament winding, Pultrusion, Mechanical characterization, Non-destructive testing, *Fabrication and Testing Methods of CFRP Composite Pipes*

## Abstract

The current work offers a comprehensive and in-depth examination of Fiber Reinforced Composite pipes mainly dealing with Carbon Fiber Reinforced Polymer (CFRP) composite pipes, with a focus on fabrication techniques, testing methodologies, diverse applications across industries, challenges, limitations, and future developments. Because of their exceptional mechanical properties, corrosion resistance, and lightweight nature, CFRP composite pipes have received a lot of attention. It delves into the various CFRP composite pipe fabrication techniques, such as filament winding and pultrusion. The review delves deeply into the multifaceted landscape of testing and characterization methodologies for CFRP composite pipes. Mechanical testing, environmental assessments, and non-destructive evaluation techniques are investigated in order to better understand their roles in determining the structural integrity and performance of these pipes. This review provides a comprehensive understanding of CFRP composite pipes, bridging the gap between research and practical implementation. This paper serves as a valuable resource for researchers, engineers, and practitioners interested in the many facets of CFRP composite pipes and their profound impact on modern industries by synthesizing knowledge from fabrication to application.

## Review Highlights

• The review paper details cutting-edge methods for fabricating CFRP composite pipes, including filament winding, pultrusion, and resin transfer moulding, emphasizing their impact on mechanical properties and structural integrity.
• It provides an in-depth analysis of various mechanical testing protocols such as tensile, compression, and flexural tests, along with non-destructive evaluation techniques like ultrasonic testing and X-ray computed tomography, to assess the performance and reliability of CFRP composite pipes.
• The paper highlights current challenges in the fabrication and testing of CFRP composite pipes, such as ensuring uniform fiber distribution and addressing interfacial bonding issues, while also discussing recent innovations and future directions to enhance material performance and application scope.

## Graphical abstract

**Figure:**
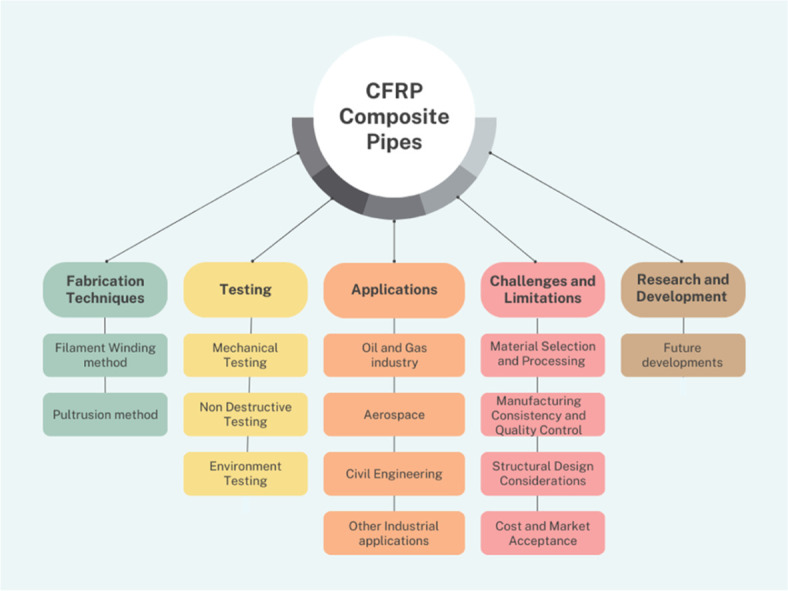

Specifications table

This table provides general information on the methodology you reviewed.

**Table:** 

Subject area:	Engineering
More specific subject area:	*Composites*
Name of the reviewed methodology:	*Fabrication and Testing Methods of CFRP Composite Pipes*
Keywords:	*CFRP composite, filament winding, pultrusion, mechanical characterization, non-destructive testing*
Resource availability:	• F.G. Alabtah, E. Mahdi, F.F. Eliyan, The use of fiber reinforced polymeric composites in pipelines: A review, Compos. Struct. 276 (2021). https://doi.org/10.1016/J.COMPSTRUCT.2021.114595. • M. Azeem, H.H. Ya, M. Kumar, P. Stabla, M. Smolnicki, L. Gemi, R. Khan, T. Ahmed, Q. Ma, M.R. Sadique, A.A. Mokhtar, M. Mustapha, Application of Filament Winding Technology in Composite Pressure Vessels and Challenges: A Review, J. Energy Storage. 49 (2022) 103,468. https://doi.org/10.1016/J.EST.2021.103468. • G. Perillo, R. Vacher, F. Grytten, S. Sørbø, V. Delhaye, Material characterization and failure envelope evaluation of filament wound GFRP and CFRP composite tubes, Polym. Test. 40 (2014) 54–62. https://doi.org/10.1016/J.POLYMERTESTING.2014.08.009. • Y. Ma, T. Sugahara, Y. Yang, H. Hamada, A study on the energy absorption properties of carbon/aramid fiber filament winding composite tube, Compos. Struct. 123 (2015) 301–311. https://doi.org/10.1016/J.COMPSTRUCT.2014.12.067. • J. Xu, Y. Ma, Q. Zhang, T. Sugahara, Y. Yang, H. Hamada, Crashworthiness of carbon fiber hybrid composite tubes molded by filament winding, Compos. Struct. 139 (2016) 130–140. https://doi.org/10.1016/J.COMPSTRUCT.2015.11.053. • P. Stabla, M. Lubecki, M. Smolnicki, The effect of mosaic pattern and winding angle on radially compressed filament-wound CFRP composite tubes, Compos. Struct. 292 (2022) 115,644. https://doi.org/10.1016/J.COMPSTRUCT.2022.115644.
Review question:	1. What are the primary fabrication techniques used for CFRP composite pipes? 2. How are CFRP composite pipes tested and characterized to ensure quality and performance? 3. What are the economic considerations and cost-benefit analyses associated with CFRP composite pipes? 4. In which industries and applications have CFRP composite pipes demonstrated significant benefits? 5. What role do government initiatives and collaborative efforts play in the adoption and standardization of CFRP composite pipes?

## Background

**Fig. 1:**
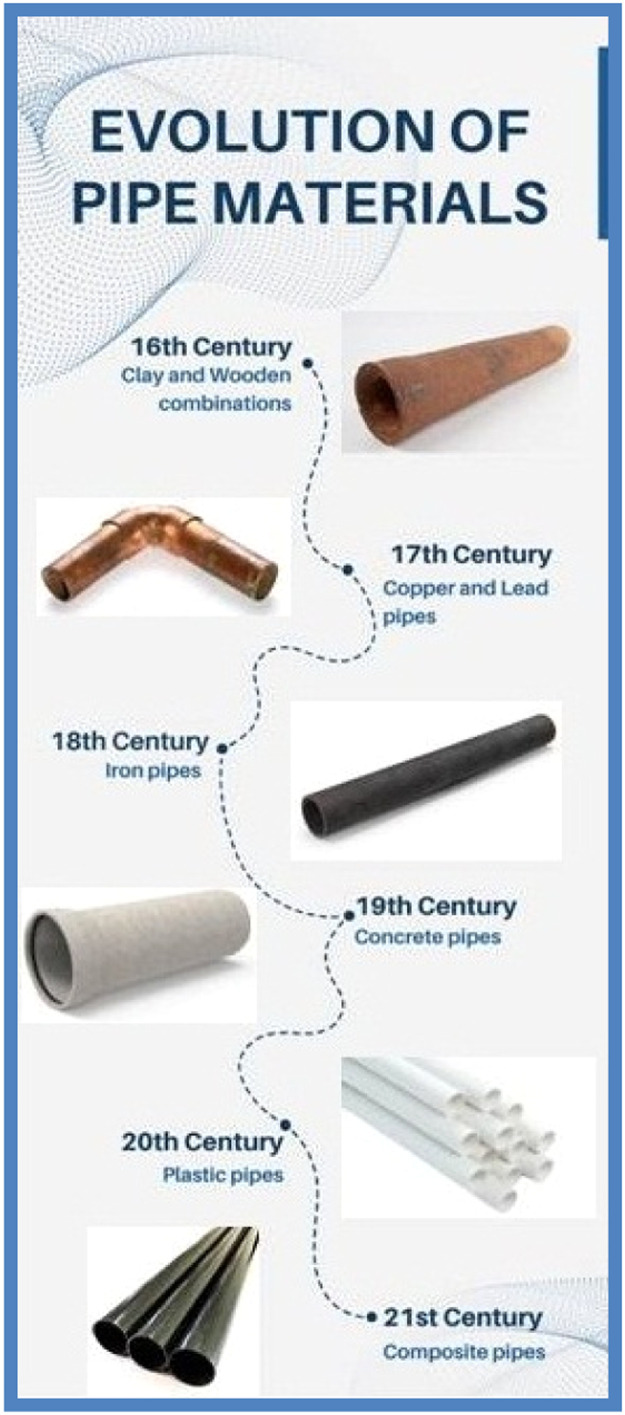
Evolution of pipe materials.

Pipe material evolution has been a continuous journey driven by technological advancement, changing industrial needs, and a growing understanding of material properties. Various materials have been used to make pipes over time, each with its own set of advantages and disadvantages [1,2]. Wood was historically one of the first materials used for pipe construction. Ancient civilizations used hollowed-out logs or wooden planks to transport water. Wood, on the other hand, was prone to decay, rot, and leakage, limiting its longevity and utility. Clay pipes emerged as an improvement over wood pipes, offering greater durability and corrosion resistance. For sewage systems and water transportation, ancient societies used fired clay pipes. Ceramic pipes were also common, especially in gravity-fed systems [3]. Metal pipes, such as copper and lead, have grown in popularity as a result of their superior strength and corrosion resistance when compared to wood and clay. Copper pipes, known for their malleability and durability, were widely used in ancient Rome for water distribution. Lead pipes, on the other hand, raised health concerns due to lead contamination [4,5]. Cast iron pipes became widely used during the Industrial Revolution. In the nineteenth century, cast iron became the standard choice for urban water supply and sewage systems due to its increased strength and durability. Cast iron, on the other hand, was heavy, prone to corrosion, and required extensive maintenance [6,7]. The rise of steel pipes in the twentieth century addressed some of the limitations of cast iron. Steel pipes provided increased tensile strength, corrosion resistance, and dependability. They found widespread application in a variety of industries, including oil and gas, water distribution, and industrial applications [8,9]. Plastic pipes, beginning with PVC (Polyvinyl Chloride) pipes, were introduced in the mid-twentieth century. Plastic pipes changed the industry because of their light weight, corrosion resistance, and ease of installation. Plastic pipes of various types, such as HDPE (High-Density Polyethylene), ABS (Acrylonitrile Butadiene Styrene), and PEX (Cross-linked Polyethylene), have gained popularity for a variety of applications [10,11]. Fibre Reinforced Polymers (FRP) and Carbon Fibre Reinforced Polymers (CFRP) are the most recent advancements in pipe materials. The strength of fibres (such as fiberglass or carbon) is combined with the versatility of polymer matrices in these materials. CFRP composite pipes have excellent strength-to-weight ratios, corrosion resistance, and durability, making them suitable for a wide range of applications, including aerospace, oil and gas, and civil engineering [12,13]. Fig. 1 represents the evolution of pipe materials over the centuries.

Carbon Fiber Reinforced Polymer (CFRP) composite pipes are a modern innovation that combines the distinct properties of carbon fiber and polymers to produce high-performance pipes for a variety of applications. CFRP composite pipes are made up of two main components: carbon fibres and a polymer matrix. Carbon fibres are extremely strong and lightweight because they are made up of thin, tightly woven carbon filaments. These fibres have been impregnated with a polymer resin, typically epoxy, which binds the fibres together and provides structural support [14,15]. Carbon fibres are well-known for their high strength-to-weight ratio. When combined with a polymer matrix, CFRP composite pipes become both lightweight and incredibly strong, allowing for efficient fluid or gas transportation while retaining structural integrity [16,17]. One of the primary benefits of CFRP composite pipes is their inherent corrosion resistance. In contrast to metallic pipes, which corrode over time due to rust and chemical reactions, CFRP pipes are unaffected by corrosive environments [18,19]. CFRP composite pipes are resistant to a wide range of chemicals, making them suitable for transporting a variety of fluids, including those that are corrosive or reactive [20,21]. CFRP composite pipe fabrication allows for intricate designs and tailored properties. Carbon fiber orientation and the polymer matrix can be customized by manufacturers to meet specific performance requirements [22,23]. Because of their resistance to environmental factors, chemicals, and mechanical stress, CFRP composite pipes have a longer service life. This increased longevity lowers maintenance and replacement costs. When compared to metals, CFRP materials have lower thermal conductivity, providing thermal insulation that aids in maintaining the temperature of the transported fluids [24,25]. Because CFRP pipes are electrically non-conductive, they eliminate concerns about electrical interference or energy losses in specific applications. CFRP composite pipes are a cutting-edge solution that takes advantage of the properties of carbon fibres and polymer matrices. Because of their corrosion resistance, chemical resistance, lightweight nature, and customizable properties, they are a versatile choice for a variety of industries looking for reliable and efficient pipe solutions [26,27].

## Method details

### Fabrication techniques for CFRP composite pipes

CFRP composite pipe fabrication techniques are critical to realizing the remarkable potential of these advanced materials. Carbon fibers’ unique properties combined with polymer matrices enable the creation of pipes with exceptional strength, corrosion resistance, and tailored characteristics. To create CFRP composite pipes that meet specific performance requirements, various fabrication methods, ranging from manual layup to automated processes, are used [28,29].

### Filament winding

**Fig. 2:**
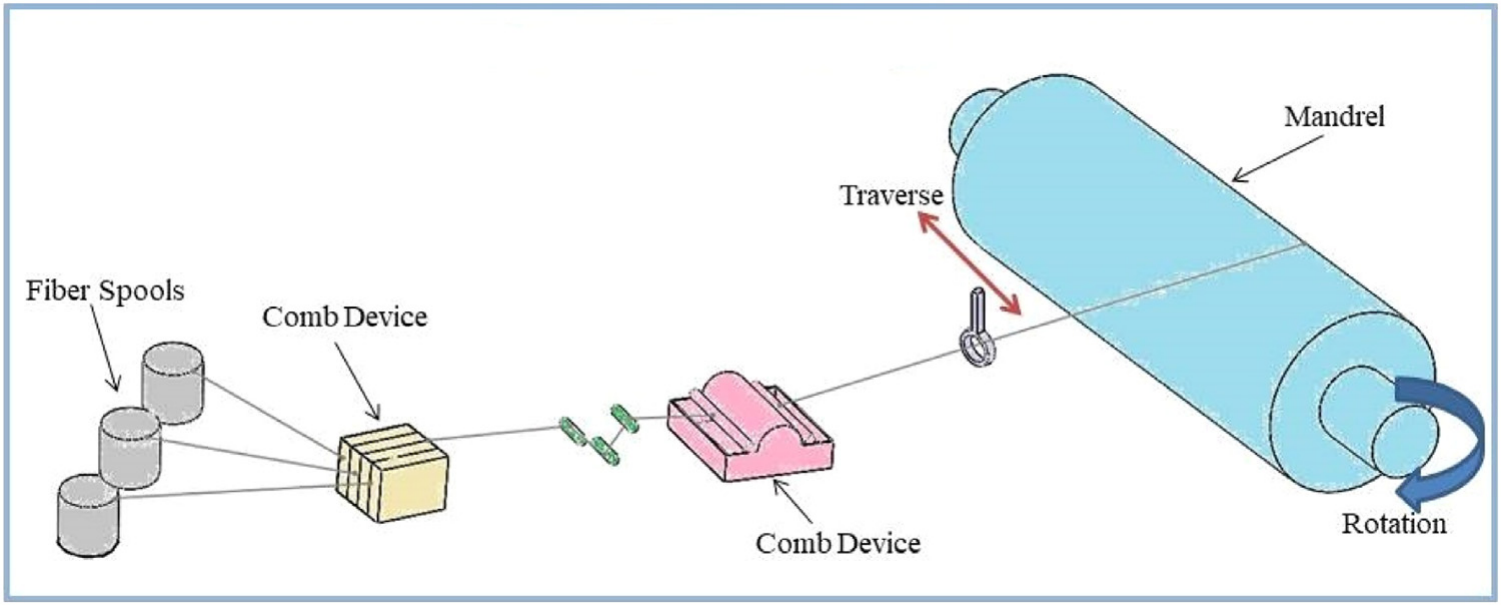
Filament winding technique schematic diagram.

The filament winding technique (Fig. 2) is an advanced and widely used method for manufacturing CFRP composite pipes. This method involves precisely winding continuous carbon fibre strands around a rotating mandrel while applying a polymer resin at the same time. The resulting composite structure has exceptional strength, durability, and tailored properties, making it suitable for a wide range of applications requiring lightweight and high-performance pipes [30,31].

The inner mold around which the carbon fibers will be wound is a mandrel, which is typically made of metal or other materials. It is meticulously prepared to correspond to the desired pipe dimensions and surface finish. The rotation and translation of the mandrel are controlled to ensure uniform winding. Continuous carbon fiber tows are fed onto the mandrel in specific patterns, often impregnated with a polymer resin. The carbon fiber orientation can be changed to optimize the pipe's mechanical properties for the intended application. Carbon fibers provide structural integrity to the pipe and contribute to its high strength-to-weight ratio. A polymer resin is applied while the carbon fibers are wound onto the mandrel. Various methods, such as spraying, dipping, or impregnation during the fiber winding process, can be used to achieve this. The resin acts as a binding agent, connecting the carbon fibers and transferring load across the composite structure. The composite structure is cured after the carbon fibers and resin is installed. The pipe is subjected to controlled temperature and pressure conditions in order to promote polymerization and hardening of the resin. The curing process ensures that a rigid and strong composite structure is formed. Some composite pipes may require post-curing after the initial curing to improve mechanical properties even further. The composite pipe is carefully removed from the mandrel after it has cured. To achieve the desired external appearance and dimensions, surface finishing processes such as sanding or coating application may be used [[32], [33], [34], [35]].

**Fig. 3:**
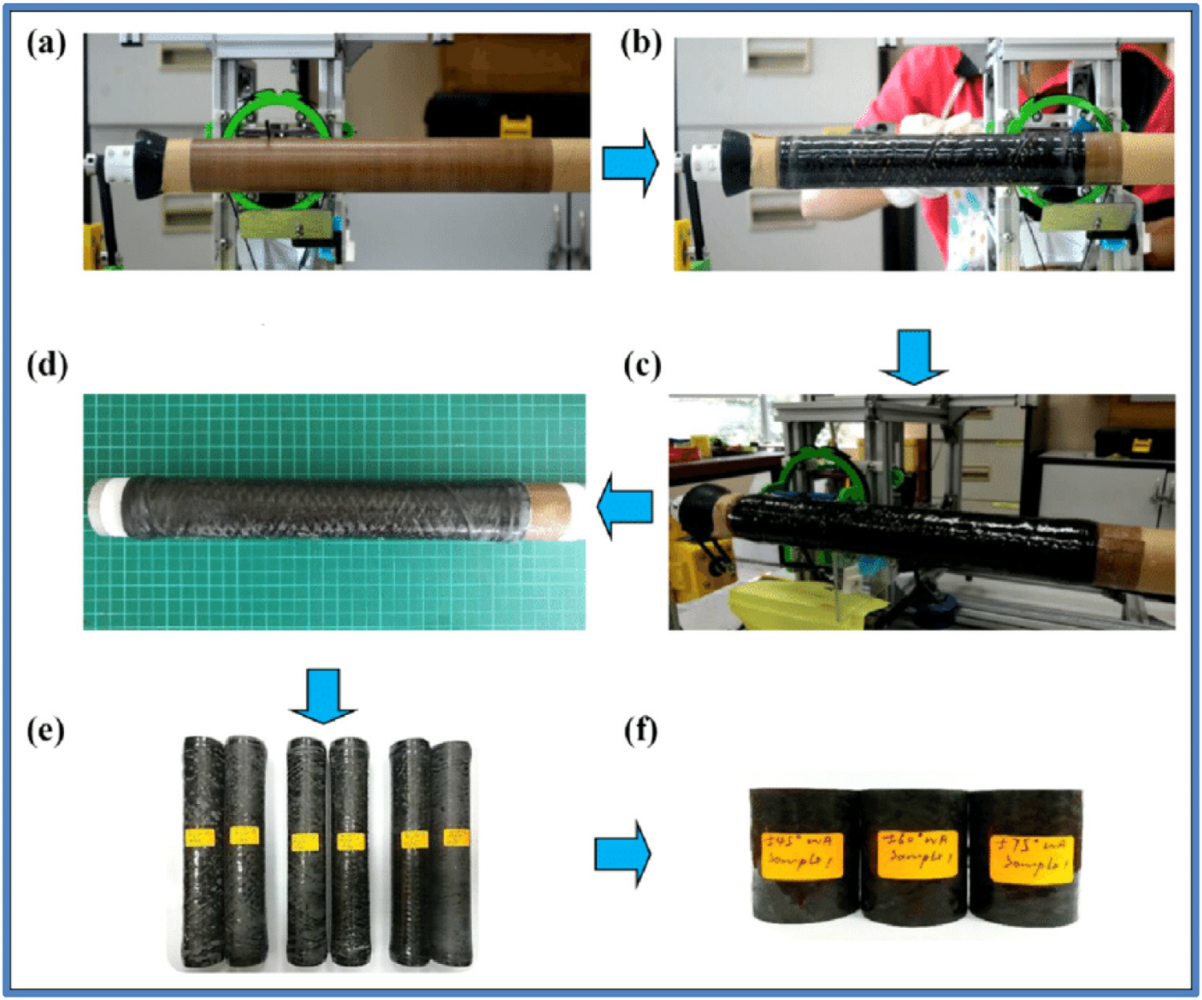
Fabrication of CFRP pipes using filament winding technique [39].

Filament winding gives you precise control over the orientation and placement of carbon fibres, allowing you to make pipes with customised mechanical properties and directional strength. The use of carbon fibres and polymer resin produces lightweight pipes with high mechanical strength, making them suitable for demanding applications. Unlike metallic pipes, CFRP composite pipes made with filament winding are naturally corrosion resistant. The filament winding process allows for the creation of pipes with complex geometries and varying diameters, allowing for a wide range of applications. Filament winding can be automated, resulting in higher quality and lower labour requirements [30,[36], [37], [38]]. Fig. 3 details the Fabrication of CFRP pipes using filament winding technique.

### Pultrusion

**Fig. 4:**
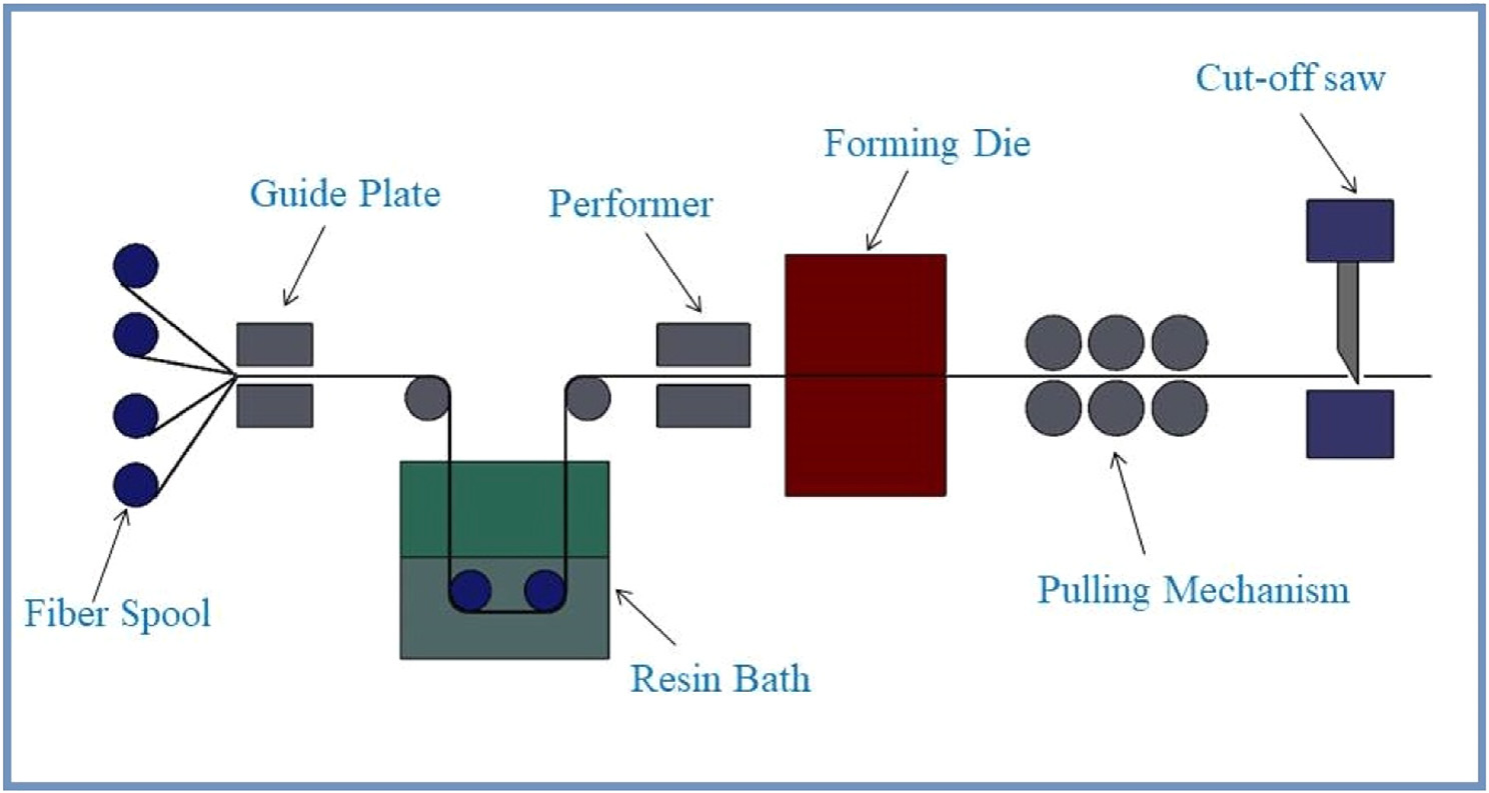
Pultrusion technique schematic diagram.

Pultrusion (Fig. 4) is a specialized method for producing CFRP composite pipes. This method involves pulling fibers continuously through a resin bath, followed by a controlled curing process. The composite material that results is then shaped and cut to form pipes with high strength-to-weight ratios, corrosion resistance, and tailored mechanical properties. Pultruded CFRP composite pipes are used in a variety of industries, including infrastructure, construction, and industrial systems [40,41]. Continuous carbon fiber rovings, which are carbon fiber bundles, are ready for the pultrusion process. Sizing agents are typically applied to these fibers to improve adhesion between the fibers and the polymer matrix. The carbon fiber rovings are drawn through a resin bath, which typically contains a thermosetting polymer resin such as epoxy. The fibers are fully impregnated as they pass through the resin, ensuring that they are coated uniformly and will bond with the polymer matrix during curing. After impregnation, the resin-soaked fibers pass through a shaping die that determines the final shape and dimensions of the pultruded composite. The die can have different profiles and features, allowing for the production of pipes with various cross-sectional geometries. After the fibers are shaped, the composite material is heated to begin the curing process. Heat causes thermosetting resin to polymerize and harden, resulting in a rigid and long-lasting structure. To achieve the desired material properties, temperature and curing time are carefully controlled. A caterpillar-like pulling system continuously pulls the cured CFRP composite material through the pultrusion process. While being pulled, the composite material passes through cutting mechanisms that trim the material to the desired length. The resulting composite pipes are inspected for defects, dimensional accuracy, and proper curing both during and after the pultrusion process. Any pipes that fail to meet the required standards are identified and removed from the manufacturing process [40,41].

Pultrusion provides a high level of process control, resulting in consistent quality and material properties across all produced pipes. The continuous arrangement of carbon fibres ensures that the resulting pipes have high strength and stiffness. Pultruded CFRP composite pipes are naturally corrosion resistant, making them suitable for environments where traditional metallic pipes would deteriorate. Pultrusion shaping dies can be customized to create pipes with a variety of profiles, allowing for design flexibility to meet specific project requirements. Pultrusion can be automated, which reduces labour requirements and ensures consistent output [[42], [43], [44], [45]].

### Resin transfer molding

Resin Transfer Molding (RTM) is a highly efficient and popular method for producing Carbon Fiber Reinforced Polymer (CFRP) composite pipes, especially when precision and high-quality finishes are required. In the RTM process, dry carbon fiber preforms, which are pre-shaped reinforcements, are placed inside a closed mold that defines the final pipe shape. The mold is then sealed, and resin is pressure-injected into the preform, allowing it to flow through and thoroughly impregnate the fibers [46]. This process ensures that the fibers are fully saturated with resin, resulting in a uniform and consistent composite material. After the resin has been injected, the mold is heated to cure the resin and solidify the composite into its final shape. One of the primary benefits of RTM is the ability to produce parts with high fiber volume fractions and excellent mechanical properties, such as high strength-to-weight ratios and superior surface finishes. Additionally, the closed-mold environment reduces resin waste and the emission of volatile organic compounds (VOCs), making RTM a more environmentally friendly option than open-mold processes. RTM is particularly well-suited to producing complex shapes and large structures with tight tolerances, making it ideal for high-performance applications in the aerospace, automotive, and industrial sectors. However, the process necessitates precise control over resin flow, mold design, and curing conditions, which can raise the initial setup costs. Despite these challenges, RTM is still the preferred method for producing high-quality CFRP composite pipes where performance and reliability are critical [47,48].

### Autoclave molding

Autoclave molding is a highly advanced and precise technique for producing Carbon Fiber Reinforced Polymer (CFRP) composite pipes that are known for their exceptional mechanical properties and low defect count. Carbon fiber layers are impregnated with resin and carefully laid up in a mold to define the shape of the pipe. This assembly is then sealed in a vacuum bag to remove any trapped air, ensuring that the composite is void-free. The vacuum-bagged assembly is placed inside an autoclave, which is a pressurized oven that applies heat and pressure at the same time. The autoclave typically operates at temperatures ranging from 120 °C to 180 °C and pressures of 7 bar or higher, depending on the resin system used. The combination of heat and pressure during curing ensures that the resin flows evenly, completely impregnates the fibers, and bonds the layers together to form a solid, uniform composite structure [49]. This process produces CFRP pipes with high fiber volume fractions, excellent strength-to-weight ratios, and superior surface finishes, making them ideal for high-performance applications in industries such as aerospace, automotive, and oil and gas. The autoclave molding process also provides precise control over fiber orientation and resin content, which can be tailored to specific performance requirements. However, the process is capital-intensive, requiring significant investment in autoclave equipment and molds, and it is relatively time-consuming due to the curing cycles involved. Despite these challenges, autoclave molding remains a gold standard in the production of CFRP composite pipes where maximum structural integrity and performance are paramount [50].

### Braiding

The braiding technique is a cutting-edge and highly efficient method for making Carbon Fiber Reinforced Polymer (CFRP) composite pipes. It is especially valued for its ability to produce parts with exceptional structural integrity and uniformity. Using a braiding machine, continuous carbon fibers are interlaced or woven into a tubular pattern. The fibers are braided over a mandrel that defines the pipe's inner diameter and shape, resulting in a seamless, highly oriented fiber structure. The braiding pattern can be customized to achieve specific mechanical properties by adjusting the angle and density of the fiber layup, resulting in optimal load distribution and increased strength. After the braiding process is completed, the braided preform is impregnated with resin, either using Resin Transfer Moulding (RTM) or other resin infusion techniques [51]. The resin is then cured, usually with heat and pressure, to solidify the composite material and bond the fibers together. The resulting CFRP pipe has exceptional mechanical properties, such as high tensile strength, impact resistance, and durability, making it ideal for demanding applications in industries such as aerospace, automotive, and oil and gas. The braiding technique has several advantages, including the ability to produce continuous and seamless pipes with consistent fiber orientation, which improves the overall structural performance of the final product. Furthermore, the braiding process is automated, resulting in high repeatability and efficiency, making it ideal for medium to high-volume production. However, the initial investment in specialized braiding equipment can be significant, and the process may be less adaptable when producing non-cylindrical or highly complex shapes. Despite these limitations, braiding remains the preferred method for producing high-performance CFRP composite pipes that require precise fiber alignment and superior mechanical properties [52].

## Testing and characterization of CFRP composite pipes

CFRP composite pipe testing and characterization are critical steps in determining the structural integrity, performance, and dependability of these advanced materials. Understanding the mechanical properties, durability, and response to various conditions of CFRP composite pipes is becoming increasingly important as their use grows in popularity across industries. This section delves into the methodologies used to rigorously test CFRP composite pipes, shedding light on the procedures used to analyse their behaviour under various loads, environments, and scenarios. Engineers and researchers gain valuable insights into the optimisation and enhancement of these high-performance pipe systems through extensive testing and characterization [[53], [54], [55]].

### Mechanical testing

Mechanical testing of CFRP composite pipes is an important process for determining structural integrity, strength, stiffness, and other mechanical properties. These tests provide critical information for design, quality control, and performance prediction, ensuring that the pipes meet safety and performance standards [61,62].

#### Tensile testing

**Fig. 5:**
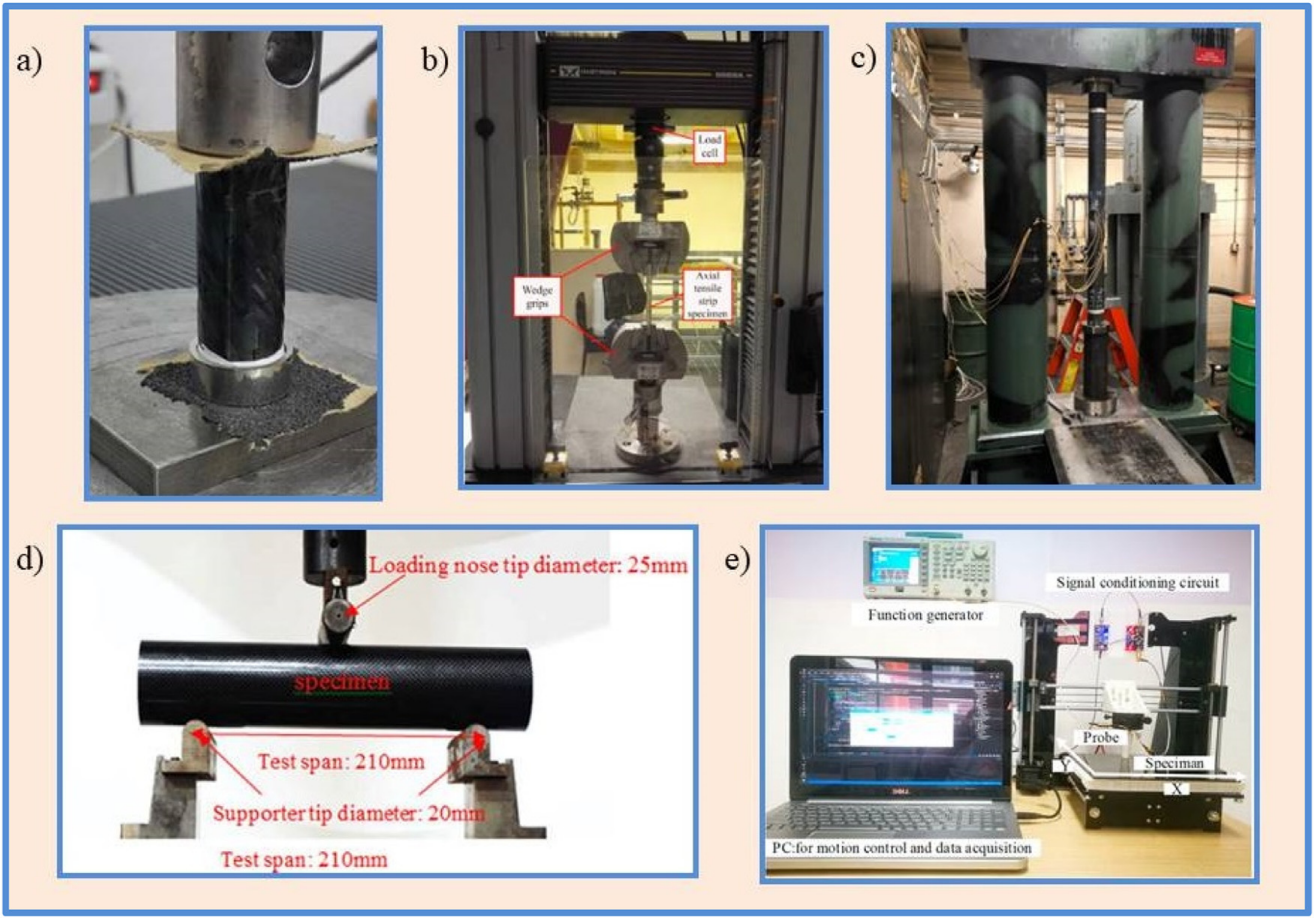
Mechanical testing of CFRP pipes (a) Compression test [56] (b) Tensile test [57] (c) Burst test [58] (d) Flexural test [59] (e) Eddy current test [60].

Tensile testing (Fig. 5b) determines a material's axial load response by pulling it until it fractures. Specimens are prepared from the pipe's wall and subjected to axial tension for CFRP pipes. This test determines the ultimate tensile strength, elastic modulus, and strain at failure of a material. It contributes to a better understanding of how the composite responds to loading along its longitudinal axis [63].

#### Compression testing

Compression testing (Fig. 5a) entails applying compressive forces to a specimen in order to determine its resistance to buckling and crushing. Compression tests are performed on cylindrical specimens extracted from CFRP pipes. This test yields information on compressive strength and stiffness [64,65].

#### Flexural testing

Flexural tests (Fig. 5d) evaluate the behaviour of a material under bending loads. Bending moments are applied to a CFRP pipe specimen using a three-point or four-point bending setup. The test determines flexural strength, flexural modulus, and deformation behaviour [[66], [67], [68], [69]].

#### Shear testing

Shear testing measures a material's resistance to internal sliding forces. Shear tests, while not as common as other tests, can provide information on the shear strength and shear modulus of the composite material [70,71].

#### Impact testing

The ability of a material to withstand sudden loading conditions is measured by impact testing. Impact tests such as Charpy or Izod can be used to evaluate the composite's resistance to impact forces. This is especially important in applications where pipes may be subjected to sudden external loads or impacts [72,73].

#### Fatigue testing

Fatigue tests simulate the repeated loading cycles that materials experience over the course of their service life. Cyclic loading of CFRP composite pipes can be used to determine how well they withstand repeated stress without failing. Fatigue tests aid in predicting the pipe's performance over time under real-world conditions [74,75].

#### Burst testing

Burst tests (Fig. 5c) simulate the pressure conditions that pipes may encounter while in use. Pipes are pressurised until they burst, allowing the ultimate pressure capacity and failure mode to be determined [74,75].

### Non-Destructive testing (NDT)

Ultrasonic testing, radiography, and acoustic emission are NDT techniques used to inspect pipes without causing damage. These methods detect internal flaws, delamination, or voids that may jeopardise the pipe's integrity [76,77].

#### Ultrasonic testing (UT)

Ultrasonic waves are sent into the CFRP pipe, and the echoes that return are analysed to determine the material's thickness, the presence of defects, and the quality of layer bonding. Internal delamination, voids, and changes in material properties can all be detected using UT [78,79].

#### Radiographic testing (RT)

RT is similar to medical X-rays in that X-rays or gamma rays are passed through the CFRP pipe. The resulting image aids in the detection of internal defects, voids, and irregularities in the structure of the composite. RT is especially useful for detecting differences in material density [80].

#### Thermography

Thermography is the process of measuring the heat distribution on the surface of CFRP pipes. Temperature changes can reveal flaws or variations in material properties. Passive thermography relies on temperature differences caused by internal defects, whereas active thermography uses external heat sources [80].

#### Acoustic emission testing (AE)

**Fig. 6:**
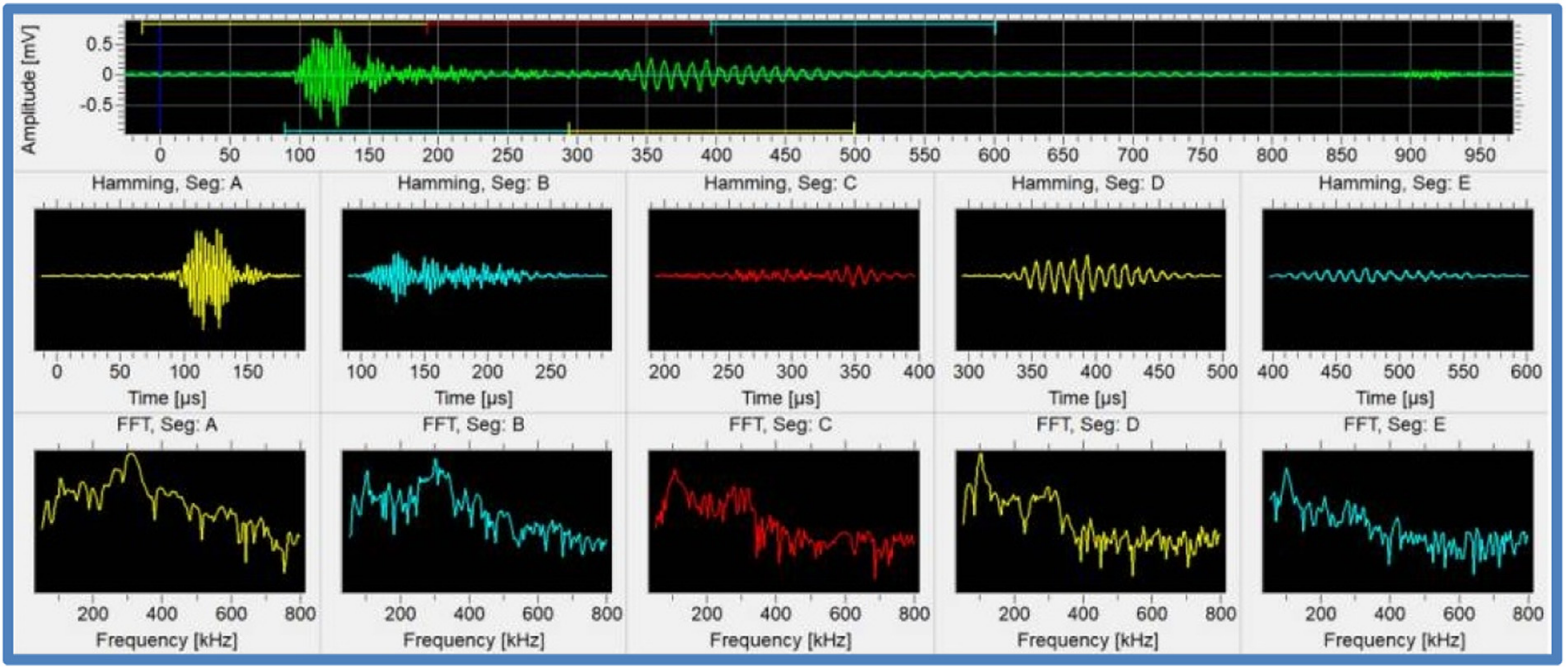
Acoustic Emission Testing frequency domain[83].

Acoustic signals generated by internal changes or defects in the CFRP composite are detected and analysed in AE. These indicators may indicate the presence of defects, delamination, or stress-related problems [81,82]. Fig. 6 indicates the generated Acoustic Emission Testing frequency domain.

#### Shearography

Laser interferometry is used in Shearography to detect surface deformations caused by internal defects or stresses in the CFRP pipe. It is especially useful for detecting delamination, disbonds, and other surface irregularities [84,85].

#### X-ray computed tomography (CT)

CT scanners, like medical CT scans, produce detailed cross-sectional images of the CFRP pipe. This method provides a comprehensive view of the internal structure, allowing for the detection of defects and delamination with high accuracy [[86], [87], [88]].

#### Impact-echo testing

The impact-echo method involves striking the surface of a CFRP pipe and analysing the resulting acoustic waves. Inspectors can detect delamination and voids by measuring the time it takes for the waves to travel through the material [89,90].

#### Eddy current testing (ECT)

ECT (Fig. 5e) detects changes in electrical conductivity or permeability within the CFRP pipe using electromagnetic induction. Cracks, voids, and other defects in conductive materials can be detected using this method [89,90].

#### Digital radiography (DR)

DR, like traditional radiography, takes X-ray images of the CFRP pipe. However, digital radiography provides better image quality and faster results, allowing for more detailed defect analysis [91].

Non-destructive testing is required to evaluate the quality and dependability of CFRP composite pipes. NDT methods ensure that pipes meet safety standards and perform as expected in various applications by detecting defects and anomalies early in the manufacturing or operational stages.

## Machinability of CFRP composite pipes

The machinability of Carbon Fiber Reinforced Polymer (CFRP) composite pipes refers to how easily these materials can be cut, shaped, or finished using various machining techniques. CFRP composites are known for their high strength-to-weight ratio, corrosion resistance, and durability; however, these same properties can make them difficult to machine when compared to traditional materials such as metals [92].

### Key challenges in machining CFRP composite pipes

Machining Carbon Fiber Reinforced Polymer (CFRP) composite pipes presents several significant challenges due to the material's distinct properties. One of the most significant challenges is tool wear and damage, as the abrasive nature of carbon fibers quickly degrades cutting tools, necessitating the use of specialized, high-hardness tools such as diamond-coated or polycrystalline diamond (PCD) tools [93]. Furthermore, the risk of delamination and fiber pull-out during machining is high, as layers of the composite may separate or individual fibers may become dislodged, jeopardizing the structural integrity and surface finish of the pipes [94]. Heat generation during machining is another critical issue, as excessive friction can cause thermal degradation of the polymer matrix, affecting the pipe's mechanical properties. The heterogeneous nature of CFRP makes it difficult to achieve a high-quality surface finish and maintain tight dimensional tolerances. This can lead to variations in surface roughness and make controlling the machining process difficult. These difficulties make it necessary to carefully choose the machining parameters, instruments, and cooling methods in order to guarantee the integrity and functionality of CFRP composite pipes [95].

### Machining techniques and considerations in machining CFRP composite pipes

To overcome the inherent challenges of Carbon Fiber Reinforced Polymer (CFRP) composite pipes, the right techniques and considerations must be chosen during the machining process. Because conventional tools can quickly deteriorate due to the abrasive carbon fibers, specialized cutting tools, such as diamond-coated or polycrystalline diamond (PCD) tools, are frequently used to minimize wear and maintain precision [96,97]. Water jet and laser cutting are examples of advanced machining techniques that are recommended because they minimize mechanical stresses on the material, which lowers the possibility of delamination and fiber pull-out. To prevent damage and guarantee a smooth finish, conventional methods like drilling and milling call for precise control over feed rates, cutting speeds, and tool geometry. In order to avoid excessive heat build-up during machining, which can degrade the polymer matrix and alter the mechanical properties of the pipe, adequate cooling and lubrication are crucial. It might also be required to apply protective coatings or polishing after machining to improve the durability and quality of the surface. When machining CFRP composite pipes, these methods and factors are essential for preserving their structural integrity and functionality [98,99].

## Applications of CFRP composite pipes

**Fig. 7:**
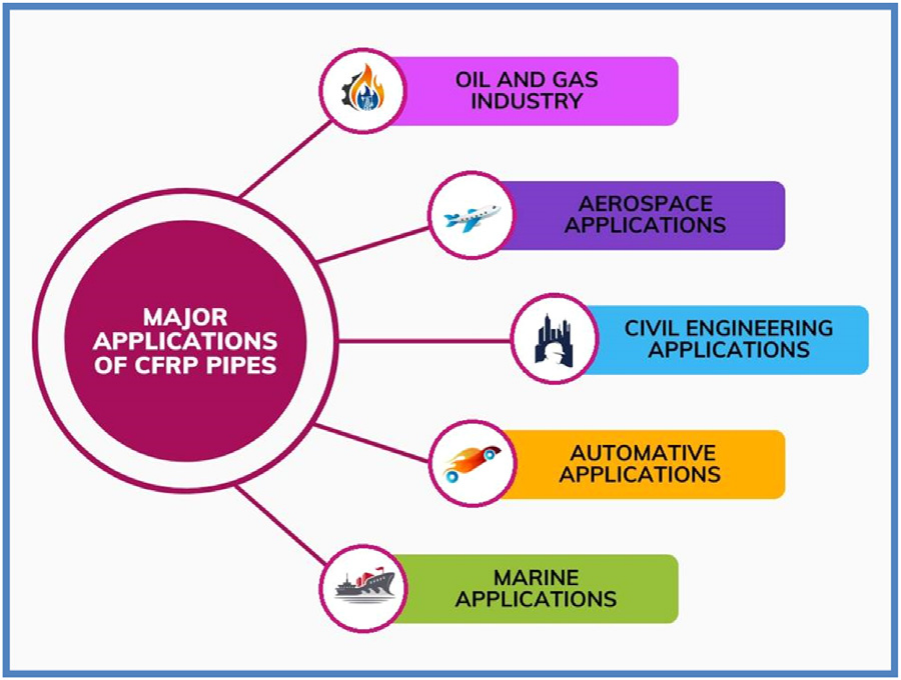
Applications of CFRP pipes.

Because of their exceptional strength-to-weight ratio, corrosion resistance, and versatility, CFRP composite pipes have found a wide range of applications (Fig. 7) across a variety of industries. These lightweight but strong pipes are designed to withstand the rigours of various harsh environments. Their uses range from aerospace and automotive to civil engineering and oil and gas exploration. Let's take a closer look at some of the most important applications for CFRP composite pipes.

### Oil and gas industry

In the oil and gas industry, CFRP composite pipes have found numerous applications (Table 1), revolutionising various processes and addressing challenges faced by traditional materials. Because of their unique combination of high strength, corrosion resistance, lightweight nature, and durability, they are ideal for a variety of functions in this demanding industry [100].

**Table 1:** Applications in O & G Industry.

Applications in O & G Industry	Description and important benefits	Ref
Oil and gas wells	Used as downhole tubing and drill pipes in oil and gas wells. Their lightweight nature reduces the load on drilling equipment, improving operational efficiency. The corrosion resistance of CFRP pipes is especially beneficial in harsh downhole environments that involve exposure to corrosive fluids and gases.	[[101], [102], [103]]
Subsea pipelines	Transport oil and gas from offshore wells to processing facilities onshore. CFRP composite pipes offer significant advantages in this application due to their corrosion resistance, minimizing maintenance and extending the service life of the pipelines. Their lightweight properties also make installation and transportation easier.	[[104], [105], [106]]
Flowlines and Riser Systems	Transport fluids from subsea wells to floating production platforms or processing facilities. CFRP composite pipes can replace traditional steel pipes in these systems, reducing weight and enhancing corrosion resistance. Additionally, in riser systems that connect subsea wellheads to platforms, CFRP pipes provide flexibility and improved fatigue resistance.	[107]
Chemical Injection Lines	Chemical injection lines are used to inject chemicals into wells to enhance oil recovery or control reservoir conditions. CFRP pipes are well-suited for this application due to their resistance to the harsh chemicals used in the oil extraction process.	[108]
Produced Water Transfer	Produced water, by-products of oil and gas production, needs to be transported and treated. CFRP composite pipes are employed in transferring produced water due to their resistance to corrosion, preventing leaks and minimizing environmental impact.	[107,108]
Offshore Platforms and Structures	CFRP composite pipes are used in various components of offshore platforms and structures, such as walkways, handrails, and access ladders. Their lightweight nature and corrosion resistance enhance safety and reduce maintenance costs.	[109]
Exploration and Drilling Tools	CFRP composite pipes are used in exploration tools, such as seismic cables, and in drilling components like casing centralizers. Their lightweight properties contribute to improved drilling efficiency and reduced wear on equipment.	[110]

### Aerospace applications

Because of their exceptional strength-to-weight ratio, durability, and corrosion resistance, CFRP composite pipes have gained significant importance in the aerospace industry (Table 2). These characteristics make them ideal for a wide range of aerospace applications where performance, weight reduction, and dependability are critical [111].

**Table 2:** Applications in Aerospace Industry.

Applications in Aerospace Industry	Description and important benefits	Ref
Aircraft Structures	Extensively used in aircraft structures, particularly for components that require lightweight yet robust materials. They find applications in wing structures, fuselage frames, tail sections, and control surfaces. Their high strength and stiffness-to-weight ratio contribute to reduced fuel consumption and improved aircraft efficiency.	[111]
Engine Components	Aircraft engines operate in extreme conditions of temperature and pressure, demanding materials with exceptional strength and resistance. CFRP composite pipes are used in engine components like thrust reverser systems, exhaust nozzles, and shrouds due to their ability to withstand these harsh environments.	[112]
Satellite and Spacecraft Structures	In space applications, where weight is a critical factor, CFRP composite pipes are used in satellite and spacecraft structures. They help reduce launch costs and provide robust support structures for instruments and equipment.	[113,114]
UAVs and Drones	Unmanned aerial vehicles (UAVs) and drones benefit from CFRP composite pipes for lightweight construction. These pipes contribute to improved maneuverability, range, and payload capacity.	[115,116]
Avionics Enclosures	Avionics enclosures, which house critical electronic systems, benefit from CFRP composite pipes due to their electromagnetic interference (EMI) shielding capabilities and lightweight design.	[117]
Antenna Support Structures	CFRP composite pipes are used to construct antenna support structures on aircraft, satellites, and space vehicles. Their lightweight nature and high strength ensure the stability and functionality of communication systems.	[118]

### Civil engineering applications

CFRP composite pipes have a wide range of applications in civil engineering (Table 3), transforming the way infrastructure is built and maintained. Because of their high strength, durability, corrosion resistance, and lightweight properties, they are useful for addressing a variety of challenges in construction and structural projects [119].

**Table 3:** Applications in Civil Engineering.

Applications in Civil Engineering	Description and important benefits	Ref
Rehabilitation of Pipelines	Rehabilitating and strengthening deteriorating or damaged pipelines. By applying CFRP liners or wraps to the interior or exterior of pipelines, their structural integrity can be restored, extending their service life and avoiding costly replacement.	[120]
Bridge Construction and Repair	They offer solutions for increasing the load-carrying capacity of bridge columns and beams, enhancing their resistance to seismic events, and rehabilitating aging infrastructure.	[121,122]
Reinforcement of Concrete Structures	CFRP composite pipes are employed to reinforce concrete structures such as beams, columns, and slabs. By bonding or wrapping CFRP materials to the concrete, the load-carrying capacity and structural performance of the concrete can be improved, extending the lifespan of the structure.	[123,124]
Seismic Retrofitting	In earthquake-prone regions, CFRP composite pipes are used for seismic retrofitting of buildings and structures. They enhance the structure's ability to withstand seismic forces and mitigate damage during earthquakes	[125,126]
Rehabilitation of Culverts and Tunnels	Applied to rehabilitate and strengthen culverts and tunnels. These pipes improve the structural integrity and load-bearing capacity of the existing structures, minimizing the need for extensive excavation and reconstruction	[127]
Structural Strengthening	Used to strengthen structural members that have experienced deterioration loads beyond the design capacity, or changes in use. This is particularly useful in historical or heritage structures where preserving the original aesthetics is important	[128]

### Other industrial applications

Aside from aerospace, oil and gas, and civil engineering, CFRP composite pipes have a wide range of industrial applications (Table 4). Their distinct combination of high strength, lightweight nature, corrosion resistance, and durability makes them useful in a variety of industries.

**Table 4:** Other Industrial Applications.

Other Industrial Applications	Description and important benefits	Ref
**Automotive Industry**	Used in the automotive sector for various applications. They find use in exhaust systems, where their lightweight properties contribute to improved fuel efficiency and reduced emissions. Additionally, CFRP pipes are employed in intake manifolds, intercoolers, and other components where weight reduction and high-performance characteristics are crucial.	[129,130]
**Marine and Offshore Applications**	In the maritime industry, CFRP composite pipes find use in applications such as seawater intake and discharge systems, ballast water treatment, and desalination plants. Their resistance to corrosion and durability in marine environments makes them an attractive choice for these applications.	[131,132]
**Electrical and Telecommunications**	Used as support structures for overhead power lines and telecommunication cables. Their lightweight and high-strength properties make them suitable for minimizing the loads on supporting towers and facilitating efficient cable installation.	[133]
**Biomedical Industry**	Employed in pharmaceutical production and processing, particularly for transporting chemical ingredients and sterile fluids. Their resistance to corrosion and contamination is essential in maintaining the quality of pharmaceutical products.	[134]

Beyond traditional industries, CFRP composite pipes have a wide range of industrial applications. Their exceptional properties make them valuable in a variety of industries, including automotive and renewable energy, as well as mining and electronics. As technology advances, the range of applications for CFRP composite pipes is likely to broaden even more.

## Challenges and limitations

While Carbon Fiber Reinforced Polymer Composite Pipes provide numerous benefits in a variety of industries, they are not without challenges and limitations. Adoption of CFRP composite pipes, like any advanced material, necessitates a thorough understanding of potential drawbacks and constraints. This section examines the challenges and limitations associated with the use of CFRP composite pipes, ranging from manufacturing complexities to cost considerations and environmental factors [135]. Addressing these challenges is essential for unlocking the full potential of these innovative materials while ensuring safe and effective utilization across diverse sectors.

In addressing the challenges and limitations of CFRP composite pipes, it is crucial to include specific statistics to provide a clearer understanding of the issues. For example, the initial cost of manufacturing CFRP pipes is significantly higher than that of traditional materials like steel, with studies showing that CFRP pipes can be up to 30–50% more expensive due to the cost of carbon fibers and specialized resins. However, this initial investment is offset by long-term savings, as CFRP pipes have a maintenance cost reduction of approximately 20–30% and a service life that can be twice as long as that of conventional pipes. Additionally, manufacturing defects such as voids and delaminations occur in about 5–10% of CFRP pipes produced through certain methods like filament winding, which can impact performance and reliability. Market adoption of CFRP pipes has also been slow, with penetration rates in industries like oil and gas remaining below 15% due to cost and technical challenges. By including such statistics, the discussion of challenges and limitations becomes more grounded, offering a more detailed and convincing analysis of the current state of CFRP composite pipes [12,136].

### Material selection and processing challenges

Material selection and processing issues are significant factors in the use of CFRP composite pipes. While CFRP has excellent properties, there are several complexities that must be addressed during the material selection and manufacturing processes. These challenges have an impact on factors such as performance, reliability, and cost-effectiveness, influencing the overall success of CFRP composite pipe projects [137,138].

It is critical for optimal performance to achieve strong adhesion between carbon fibres and the polymer matrix. Delamination, reduced mechanical properties, and premature failure can all result from poor fiber-matrix interaction. To address this issue, effective bonding techniques and the selection of appropriate matrix materials are required [139,140]. The resin system chosen affects the mechanical properties, durability, and resistance to environmental factors of the pipe. Controlling the curing process and ensuring proper resin selection are critical for achieving consistent and desired material characteristics [141,142]. Maintaining consistency and uniformity in the manufacturing process is critical for producing CFRP composite pipes with predictable properties. Inconsistencies in material behaviour can be caused by variations in fiber orientation, resin distribution, and curing conditions. Strict inspection and testing procedures are used to ensure the quality of CFRP composite pipes. It is critical to detect defects, voids, or anomalies during manufacturing and processing to avoid compromising structural integrity [143,144]. Material anisotropy, fiber orientation, and load distribution are all factors to consider when designing CFRP composite pipes. Developing optimal designs that take advantage of the unique properties of CFRP while accounting for manufacturing constraints can be difficult [145,146]. Due to differences in material properties and structural behavior, joining CFRP composite pipes to other materials or repairing damaged sections can be difficult. It is critical to develop dependable joining techniques and repair methods in order to keep the pipes in good condition [147]. CFRP composite pipe manufacturing requires energy-intensive procedures and the use of chemical resins. Balancing the benefits of CFRP with environmental concerns and sustainability goals is a difficult task that must be approached with caution [[148], [149], [150]]. When compared to traditional materials such as steel or concrete, the cost of CFRP composite pipes may be higher. Balancing the improved performance of CFRP with the cost implications can be difficult, especially for projects with limited budgets. End-of-life considerations present difficulties in recycling or disposing of CFRP composite pipes. Creating efficient recycling methods to manage waste while minimizing environmental impact is a continuous challenge [[151], [152], [153]]. Working with CFRP materials necessitates the application of specialized knowledge and skills. Maintaining quality and safety requires ensuring that manufacturers, engineers, and technicians have the necessary expertise to handle CFRP composite pipes [151]. To address these material selection and processing challenges, material scientists, engineers, manufacturers, and researchers must work together. The industry can overcome these limitations by developing innovative solutions and refining manufacturing techniques, maximizing the benefits of CFRP composite pipes in various applications.



### Manufacturing consistency and quality control

When producing CFRP composite pipes, manufacturing consistency and quality control are critical. Maintaining stringent quality control measures and ensuring manufacturing uniformity are critical for producing pipes with reliable mechanical properties, durability, and safety [154].

It is difficult to maintain consistent and accurate fiber orientation throughout the pipe. Variations in fiber alignment can result in unequal load distribution and poor mechanical performance [155]. It is critical for structural integrity to ensure uniform impregnation of carbon fibers with resin. Inadequate resin distribution can lead to voids, weak spots, and decreased strength [156]. To achieve optimal polymerization of the resin matrix, precise control of curing temperature and time is required. Inadequate curing can result in incomplete curing or material deformations [157]. The smoothness and uniformity of the pipe's outer surface is critical for both aesthetics and performance. Surface imperfections can have an impact on fluid flow, corrosion resistance, and structural integrity [158,159].

It is critical to develop standardized manufacturing procedures with well-defined parameters and controls. Consistency can be improved by optimizing parameters such as resin mixing ratios, curing cycles, and fiber layup techniques [[160], [161], [162]]. Automated manufacturing processes, such as automated fibre placement (AFP) or filament winding machines, can reduce human error while improving repeatability [163,164]. When in-line inspection techniques are used during manufacturing, defects or inconsistencies can be detected and addressed immediately. Voids or irregularities can be detected using ultrasonic or optical inspection techniques. Regular sampling and testing of pipes throughout the manufacturing process aids in ensuring quality standards are met. Mechanical properties can be assessed using tensile, compression, and flexural tests, while non-destructive testing methods can detect internal flaws [93,165].

### Structural design considerations

When in-line inspection techniques are used during manufacturing, defects or inconsistencies can be detected and addressed immediately. Voids or irregularities can be detected using ultrasonic or optical inspection techniques. Regular sampling and testing of pipes throughout the manufacturing process aids in ensuring quality standards are met. Mechanical properties can be assessed using tensile, compression, and flexural tests, while non-destructive testing methods can detect internal flaws [166,167]. Understanding the mechanical properties of CFRP materials, such as tensile strength, stiffness, and fatigue behaviour, is critical for successful design. These characteristics guide the selection of appropriate materials and assist in ensuring that the pipes meet specific performance requirements. It is critical to analyse the expected load conditions, which include axial, bending, torsional, and pressure loads. The structural integrity of the pipe is ensured by designing it to withstand these loads while taking stress concentration points into account. Buckling analysis is essential for avoiding premature failure caused by compressive loads. Buckling problems can be avoided by taking into account factors such as pipe geometry, end conditions, and lateral support. FEA software allows engineers to perform in-depth structural analysis, allowing them to simulate various load scenarios, assess stress distribution, and optimise designs for performance and safety [168,169].

### Cost and market acceptance

Carbon Fiber Reinforced Polymer composite pipes' cost and market acceptance are critical factors influencing their adoption and success in various industries. While CFRP pipes have numerous advantages, their cost and the market's willingness to accept these products are important factors to consider [170].

CFRP materials are manufactured using complex processes and expensive raw materials such as carbon fibers and specialized resins. These expenses can add to the overall cost of CFRP pipes. Due to differences in material properties and manufacturing methods, industries that have traditionally used conventional materials such as steel or concrete may face higher initial investment costs when switching to CFRP composite pipes. When weighing the long-term benefits of CFRP composite pipes in terms of reduced maintenance, longer service life, and improved efficiency, higher upfront costs may be justified. A comprehensive analysis of the total lifecycle costs, including installation, operation, maintenance, and replacement, aids in demonstrating the long-term cost-effectiveness of CFRP pipes. Demonstrating how the unique properties of CFRP composite pipes can lead to increased operational efficiency, decreased downtime, and increased productivity can make them economically viable options. Sharing real-world case studies and success stories of projects that have benefited from CFRP composite pipes can aid in the development of market credibility and trust. Government initiatives that promote the use of advanced materials, such as CFRP composite pipes, through grants, subsidies, or tax breaks, can help to drive market adoption [171].

## Research and development in the field

Carbon Fiber Reinforced Polymer Composite Pipes Research and Development (R&D) is a dynamic and essential endeavor that drives innovation, pushes boundaries, and unlocks new possibilities across various industries. Continuous R&D efforts play a critical role in refining manufacturing techniques, optimizing material properties, enhancing performance, and addressing challenges associated with CFRP composite pipes as technology advances and demands evolve. This section delves into the on-going research and development efforts that contribute to the evolution and advancement of CFRP composite pipes, shaping their potential to revolutionise industries and address critical global challenges. Carbon Fibre Reinforced Composites research and development activities Polymer composite pipes are critical in advancing technology, improving performance, and addressing issues associated with these novel materials. Continuous R&D efforts contribute to the evolution of CFRP composite pipes, enabling new applications and increasing competitiveness in a variety of industries [172].

## Conclusion

Reinforced with carbon fibre Polymer composite pipes are at the forefront of technological advancement, providing a combination of strength, durability, and versatility that outperforms traditional materials. These pipes have demonstrated their potential to revolutionize industries and redefine structural solutions in fields ranging from aerospace to civil engineering, oil and gas to automotive. The journey through this exploration has revealed the breadth and depth of applications for CFRP composite pipes. Their incorporation into aerospace has resulted in lighter, more efficient aircraft, while in civil engineering; they have increased the resilience of bridges and pipelines. The oil and gas industry has taken advantage of their corrosion resistance and high-performance capabilities, while the automotive industry seeks to use them to improve fuel efficiency. While the benefits are obvious, there are still challenges. Manufacturing complexities, market acceptance, and cost considerations necessitate collaborative R&D efforts. The pursuit of advanced manufacturing techniques, material innovations, and sustainable practises demonstrates the commitment to overcoming obstacles and realising the full potential of CFRP composite pipes. As technological boundaries continue to expand, so do the opportunities for CFRP composite pipes. Industry, researchers, and policymakers working together pave the way for standardisation, regulation, and widespread adoption. We can harness the transformative power of CFRP composite pipes through collaborative efforts, ensuring a more resilient, efficient, and sustainable future across a wide range of applications.

## Ethics statements

The paper reflects the authors' own research and analysis in a truthful and complete manner.

## CRediT authorship contribution statement

**Senthil Maharaj Kennedy:** Conceptualization, Methodology, Software, Writing – original draft. **R.B. Jeen Robert:** Supervision. **R. Malkiya Rasalin Prince:** Writing – review & editing. **G.S. Hikku:** Visualization. **M. Kaliraj:** Data curation, Investigation.

## Declaration of competing interest

The authors declare that they have no known competing financial interests or personal relationships that could have appeared to influence the work reported in this paper.

## Data Availability

No data was used for the research described in the article.
